# Plasmid-Mediated Transfer of Antibiotic Resistance Genes in Soil

**DOI:** 10.3390/antibiotics11040525

**Published:** 2022-04-14

**Authors:** Miaoling Meng, Yaying Li, Huaiying Yao

**Affiliations:** 1 Research Center for Environmental Ecology and Engineering, School of Environmental Ecology and Biological Engineering, Wuhan Institute of Technology, Wuhan 430073, China; miaolingmeng0811@163.com; 2 Key Laboratory of Urban Environment and Health, Ningbo Observation and Research Station, Institute of Urban Environment, Chinese Academy of Sciences, Xiamen 361021, China; yyli@iue.ac.cn; 3 Zhejiang Key Laboratory of Urban Environmental Processes and Pollution Control, CAS Haixi Industrial Technology Innovation Center in Beilun, Ningbo 315830, China

**Keywords:** plasmid, antibiotic resistance genes, gene transfer, soil

## Abstract

Due to selective pressure from the widespread use of antibiotics, antibiotic resistance genes (ARGs) are found in human hosts, plants, and animals and virtually all natural environments. Their migration and transmission in different environmental media are often more harmful than antibiotics themselves. ARGs mainly move between different microorganisms through a variety of mobile genetic elements (MGEs), such as plasmids and phages. The soil environment is regarded as the most microbially active biosphere on the Earth’s surface and is closely related to human activities. With the increase in human activity, soils are becoming increasingly contaminated with antibiotics and ARGs. Soil plasmids play an important role in this process. This paper reviews the current scenario of plasmid-mediated migration and transmission of ARGs in natural environments and under different antibiotic selection pressures, summarizes the current methods of plasmid extraction and analysis, and briefly introduces the mechanism of plasmid splice transfer using the F factor as an example. However, as the global spread of drug-resistant bacteria has increased and the knowledge of MGEs improves, the contribution of soil plasmids to resistance gene transmission needs to be further investigated. The prevalence of multidrug-resistant bacteria has also made the effective prevention of the transmission of resistance genes through the plasmid-bacteria pathway a major research priority.

## 1. Introduction

Antibiotics promote healthcare and animal husbandry by inhibiting the growth and reproduction of microorganisms and by treating and preventing bacterial infections. However, the chronic use of large amounts of antibiotics can create selection pressures that cause resistant bacteria to develop resistance genes (ARGs). ARGs are widespread in clinical settings, human hosts, plants, animals, and in virtually all natural environments [1,2,3,4].

Vertical gene transfer (VGT) and horizontal gene transfer (HGT) are the main methods by which ARGs proliferate and spread in host bacterial cells. HGT, which includes transformation, splicing and transduction, is a method of transferring genetic material such as resistance genes, between conspecifics or different species of bacteria via mobile genetic elements (MGEs), rather than by reproductive processes. MGEs include transposons, integrons, phages, plasmids, etc. These critical MGEs may facilitate the spread of multidrug resistance. Plasmids carry a wide range of drug resistance genes, such as *tet*, *qnr* variants [5], *aac(6’)-lb-cr*, and the efflux pump genes *oqxAB* and *qepA*, and are the major vector for HGT. HGT is the main mechanism for the production and spread of ARGs and drug-resistant bacteria in the environment [6,7,8]. Chen et al. [4]. identified the dynamic migration of the *intI* and *sul* genes between water and sediment, with *intI* being closely associated with some specific genes in sediment. *intI* is present in the vast majority of bacteria and contributes to the transfer of ARGs in soil.

Soils play a vital role in the healthy functioning of the biosphere and in the continuation of the human race [9]. However, the current epidemic of antibiotic resistance in soil is an urgent environmental issue affecting human health worldwide. For example, in China, ensuring soil health is an important strategic goal for sustainable development. Exploring the plasmid-mediated transfer of ARGs in soil is important for soil antibiotic resistance and ensuring soil safety. This paper reviews the coupling mechanism of plasmids and plasmid-mediated transfer of resistance genes in the soil environment and lays the foundation for further experimental studies.

## 2. Comparison of Plasmid Extraction and Analysis Methods

Plasmid isolation is usually performed using endogenous culture methods from the host or by independent isolation methods based on plasmid-encoded traits. Plasmid extraction is used to isolate plasmids from bacterial genomic DNA, remove impurities such as proteins and RNA, and obtain relatively pure plasmids, such as by alkaline lysis. Alkaline lysis is the most widely used method for preparing plasmid DNA. Chromosomal DNA is denatured in an alkaline environment and is not easily renatured. Plasmid DNA can be separated from chromosomal DNA because it has a ring-like structure and can be renatured more quickly under neutral conditions. Agarose gel electrophoresis assists in the detection of plasmid DNA in bacterial DNA extracts, but the subsequent isolation and purification of plasmids is very difficult. Methods to extract plasmids from complex environments are quite limited. The existing general commercial plasmid DNA purification kits are not suitable for environmental samples and often cause chromosomal DNA contamination [10]. The transposon-aided capture system (TRACA) for plasmids facilitates the isolation of resistance gene-encoding plasmids from samples of complex composition. In this system, genomic DNA is cut with DNase, and plasmids with transposons carrying replication start sites and selectable markers are captured. This method is ideal for isolating plasmids with small copy numbers but does not capture linear plasmids and may even yield the wrong total number of plasmids. Jones et al. [11] used TRACA to obtain plasmids from metagenomic DNA extracts and stably maintain them in surrogate hosts. Plasmids isolated using TRACA have traits that are independent of the plasmid encoding them, such as selectable markers, host species mobilization traits, and the ability to replicate in host species. This means that even if the plasmid lacks the traditional selectable markers, it can still be isolated from Gram-negative (G−) and Gram-positive (G+) bacteria using TRACA and maintained in *Escherichia coli*. Metagenomics-based extraction and sequencing approaches also have limitations. An insufficient sequencing depth usually makes it difficult to extract intact plasmid sequences from the data. Additionally, genes in low abundance are easily lost due to their small fragment size and difficulty in assembly. In addition, plasmids usually contain repetitive sequences compared to genomic DNA, which makes the formation of short-read data challenging. Exogenous plasmid isolation [12] allows the isolation of linear plasmids using recipient bacteria to capture plasmids directly from parental crosses of complex samples. However, this method is highly dependent on the stability of the plasmid in the host and the binding of the plasmid in the sample. Currently, multiplex displacement amplification (MDA) based on metagenomics analysis is widely used.

The MDA method is a process in which all bacterial genomic DNA is removed from the total DNA sample using a plasmid-safe DNA enzyme, and the rest of the cyclic plasmid DNA is amplified and detected by a phi29 DNA polymerase with a loop-rolling mechanism. This method amplifies all the extracted circular plasmid DNA and produces a large amount of plasmid DNA, regardless of the number of plasmids. However, as with TRACA, linear plasmids are not separated by this method. Plasmids with larger copy numbers are easily degraded by DNase to form smaller fragments during the extraction process. Nucleotides on short-loop plasmids can be copied each time they bind to polymerase. By this method, Kav et al. [13] isolated and purified total bovine rumen plasmid DNA and performed deep sequencing using Illumina technology. The improved plasmid purification method can also be used to obtain plasmids from other ecological sites and to analyse the plasmid population in a nonculture mode using deep sequencing and metagenomic approaches [10].

Plasmid metagenomic analysis contributes to the understanding of the structure and function of the environmental plasmid community. It identifies the sites of plasmid enrichment and the additional genetic elements of the plasmid based on the environmental sample from which the plasmid was obtained. Li et al. [14] used a combination of MDA and pyrophosphate sequencing to construct a microbial library and performed experiments with existing gene libraries for a comparative analysis. The method has not been fully optimized because steps such as nucleic acid exonuclease treatment and whole-gene amplification favour small and gap-free plasmids. Jrgensen et al. [15] proposed a method with which to identify intact small plasmids from a genome-wide shotgun sequencing macrogenomic dataset. A total of 616 loop sequences were identified in the rat caecum, of which 160 genes had plasmid replication domains. In silico plasmid identification was on the Illumina platform is extremely successful (95%), with minimal risk of in vitro false positives.

## 3. Plasmid Transfer Mechanisms

**Figure 1:**
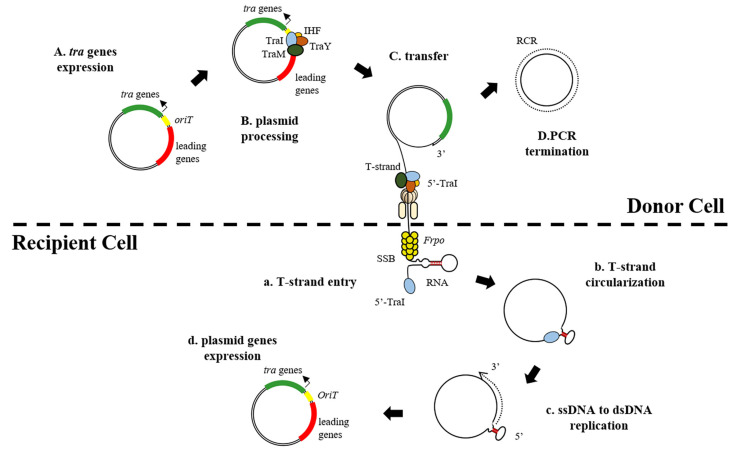
Schematic diagram of the splice transfer of plasmids from donor cells to recipient cells. The *tra* regions encode all genes involved in conjugational transfer (green); the origin of transfer *oriT* (yellow); the leading gene (red) is the first to be transferred into the recipient cell; Other Tra proteins (TraI, TraM, and TraY) constitute the relaxosome, which, in combination with the integration host factor (IHF), binds to *oriT*; chromosomal single-strand binding protein SSB; the leading region contains a specific 328 bp Frpo region (for F plasmid RNA polymerase).

Splice transfer is the process of plasmid exchange between bacteria through direct or indirect contact. Plasmids can carry ARGs to the recipient cell, thus facilitating the transfer of antibiotic resistance (Figure 1). More than 50% of plasmids are available for transfer by splicing [16]. ARG-carrying MGEs have been widely reported in a variety of settings. Coupled plasmids usually carry all the genes required for transfer. These genes encode different modules or functions.

A growing number of studies on plasmid isolation and sequence analysis have indicated great diversity in the genetic characteristics and structures of plasmids. This diversity suggests that different plasmids may use different regulatory systems, molecular responses or strategies to accomplish gene transfer. Splicing can occur between identical bacterial species or between unrelated groups at large taxonomic distances [17]. Environmental factors play an essential role in plasmid splicing efficiency. In sludge sediment, *Pseudomonas*, *Actinobacter*, *Enterobacter* and *Aeromonas* are known to be the most metastable genera [18,19,20].

### 3.1. Within the Donor Cell

To splice the donor strain, the transfer gene first needs to be expressed and aggregated in the transposable zone of the plasmid. Plasmids encode all of the type IV secretion system (T4SS)-binding-related protein factors required for pair formation as well as the relaxation component required prior to transfer. Prior to DNA transfer, protein complexes (relaxosomes) begin to assemble and carry out activities. Other Tra proteins form the relaxosome (TraI, TraM and TraY), which binds to the integrated host factor (IHF) on oriT and is transferred via the cleavage reaction of TraI relaxase. The TraI relaxase protein catalyses the nicking reaction, leading to a relaxation of plasmid dsDNA. After the nicking reaction, cyclic ssDNA in the donor is turned into dsDNA by rolling circle replication (RCR), at which point the linearized T-stranded DNA combined with TraI at the 5’ end enters the recipient cell via the conjugated pore. Briefly, the interaction of relaxosome with the type IV coupling protein (T4CP) initiates the transfer of the protein.

Furthermore, T4CPs are DNA-dependent ATPases that are fixed on the cell membrane through the N-terminal structural domain. Membrane-anchored T4CPs interact directly with relaxors to form a hexameric structure on the T-chain that is actively translocated through the coupling pore during transfer. The RCR is fundamental to the conjugation plasmid transfer process in many bacteria. In the spliced plasmid, the RCR reaction is carried out by the relaxase protein. It achieves RCR initiation mainly by cleaving double-stranded DNA at the double-stranded origin (dso) or oriT site [5]. Notably, the replication of the two ssDNA strands occurs in different cells, whereby the leading strand is replicated in the donor cell, while the trailing strand (T-strand) is replicated in the recipient cell. In the recipient, ssDNA is converted to dsDNA by RCR in the donor while the TraI-bound T-strand is transferred.

### 3.2. Within the Receptor Cell

The relaxosome is moved to the receptor, where it is refolded and primed to undertake the physiological tasks required for the splice transfer process. The pull of the relaxase from the acceptor and the push of the T4CP from the donor may facilitate the passage of the T-strand through the conjugate pore. Once the ends of the acceptor are joined together, the relaxase performs the ligation reaction, leading to recirculation of the ssDNA plasmid. Upon entry into the receptor, the T-strand of ssDNA is wrapped by the single-stranded binding protein (SSB) of the host chromosome. The single-stranded promoter *Frpo* has a stem–loop structure that can be identified by host RNA polymerase to trigger the synthesis of RNA primers. In other words, *Frpo* assists in initiating DNA synthesis reactions and early gene expression. After the T-strand enters the recipient cell, ssDNA is converted to dsDNA. Once ssDNA is converted to dsDNA, the transferred plasmid genes are expressed in recipient cells. The phenotype of the recipient cell is thus transformed into a transconjugant with additional metabolic properties.

## 4. Plasmid-Mediated Transfer of Antibiotic Resistance Genes

An MGE identified in a bacterial strain in 2003 was one of the first indicators of the existence of antibiotic resistance [21]. Since then, bacterial strains with resistance to ampicillin, chloramphenicol, erythromycin, streptomycin and tetracycline have been found in frozen soil samples [22,23]. Antibiotic resistance genes are widely present in a variety of environments, whether natural without human intervention or heavily contaminated with antibiotics (Table 1). The well-known dominant phyla in soil are *Proteobacteria*, *Acidobacteria*, *Actinobacteria*, *Verrucomicrobia*, *Bacteroidetes*, *Chloroflexi*, *Gemmatimonadetes* and *Firmicutes* [24]. A recent study has found that drug-resistant bacteria such as *Actinobacterium*, *Bacillus*, *Xanthobacteraceae* and *Geobacter* species, are common latent hosts for multidrug resistance genes (MRGs) [25]. Polymyxins have therefore been repurposed for infections caused by multidrug-resistant Gram-negative bacteria [26]. Colistin possesses antibacterial activity against members of the *Enterobacteriaceae* family, including *Klebsiella* species, *Escherichia coli* (*E. coli*), *Shigella* species, *Enterobacter* species, and *Salmonella* species [27]. The main pathway through which bacteria obtain external ARGs and develop resistance is HGT. HGT mainly occurs through transformation, splicing and transduction [28]. The horizontal transmission of ARGs among bacteria is primarily driven by bacterial plasmids, which facilitate the transfer of resistance genes. ARGs such as those encoding broad-spectrum β-lactamases (ESBLs) (e.g., CTX-M), carbapenemases (e.g., KPC, NDM, and OXA-58) [29], and mucilage resistance (e.g., MCR-1) [30], are prevalent in Gram-negative bacteria. Several Gram-negative bacteria, such as *Pseudomonas*, *Acinetobacter* and *Stenotrophomonas* species isolated by Kudinova et al. [31] have simultaneously developed resistance to multiple antibiotics. Plasmids were also detected in some dominant Gram-positive bacteria, such as *Bacillus*, *Microbacteriaceae*, and *Methanobacterium* species, suggesting that ARGs are highly likely to be transferred in both G− and G+ bacteria [32].

### 4.1. Presence of ARGs in the Natural Environment

ARGs are ubiquitous in the natural environment. On the one hand, they originate from the production of antibiotics or their derivatives by microorganisms in the soil. On the other hand, biological interactions between bacteria and other microorganisms, such as antagonistic interactions between fungi and bacteria, affect bacterial community composition and the abundance of ARGs directly [51].

ARGs have been found to be present in most terrestrial ecosystems on Earth with no or limited anthropogenic disturbance, including seabed, primeval forests, and even polar regions. Inka et al. [33] identified three sulfonamide-resistant synthases in beech and pine forest soils with different taxonomic origins. This suggests that sulfonamide antibiotic resistance occurs naturally in bacterial communities in forest soil. Song et al. [34] detected a large number of ARGs resistant to modern antibiotics in soils of primary forests in China with very low levels of antibiotics in the soil, indicating that forest soils are highly likely to be a source of potential resistance traits. The low abundance of MGEs in forest soils and their nonpositive association with ARGs reflect the minimal likelihood of HGT in forest soil environments. Kim et al. [39] detected a total of 70 independent ARGs related to 18 antibiotics in the Arctic permafrost zone using a macrogenomic approach. The genomes of permafrost and clinical strains contain similar mobile elements and prophages [52], suggesting that strains in the natural environment exhibit an extremely strong horizontal transfer of genetic material. Permafrost strains, although related to various clinical isolates, do not form separate clusters in the phylogenetic tree. Belov et al. [53] analysed the macrogenomes of perennial permafrost and sediments; *Proteobacteria*, *Firmicutes*, *Chloroflexi*, *Acidobacteria*, *Actinobacteria* and *Bacteroidetes* were the most common taxa, and the bacterial abundance was high in the microbial communities of the Canadian Arctic. Paun et al. [54] obtained and identified the first strains of bacteria from 13,000-year-old ice cores that accumulated in caves over many years since the Late Ice Age. Among the isolated bacteria, Gram-negative bacteria were more resistant than Gram-positive bacteria. Over 50% of the strains showed high resistance to 17 antibiotics. Some of these strains can inhibit the growth of typically clinically resistant strains, revealing a metabolic profile with potential applications. Mootapally et al. [40] evaluated antibiotic resistance groups in pelagic sediments and found that the dominant genes *carA*, *macB*, *bcrA*, *taeA*, *srmB*, *tetA*, *oleC* and *sav1866* were mainly resistant to macrolides, glycopeptides, and tetracyclines. Nathani et al. [41] studied a pelagic sediment microbiome for marine resistance groups and their corresponding bacterial communities. A total of 2354 unique resistance genes were identified in a comparison with samples from the open Arabian Sea, showing the presence of *tlrC* genes in addition to *carA*, *macB*, *bcrA*, *taeA*, *srmB*, *tetA*, *sav1866* and *oleC*. Moreover, *Proteobacteria*, *Actinobacteria* and *Bacteroidetes* were the predominant phyla in the deep-sea sediments.

### 4.2. Prevalence and Spread of ARGs under Antibiotic Selection Pressure

#### 4.2.1. Transfer of ARGs from Severely Contaminated Sites

Antibiotics have been extensively used in healthcare and farm animal husbandry to treat or prevent bacterial infections and promote animal husbandry. However, the overuse of antibiotics has led to antibiotic residues in clinical settings and in soil on farms, sewage treatment plants, and other sites. These residues are potentially toxic to organisms, resulting in the enrichment of ARGs, making it an emerging and persistent environmental pollutant [55]. Hospitals consume large amounts of antibiotics, especially β-lactams, quinolones and methotrexate [56]. However, their residues in hospital wastewater are unknown. The efficiency of antibiotic removal from hospital wastewater treatment processes was reported to be 74–81% [57]. Among various types of antibiotics, the removal efficiency for β-lactam antibiotics was high (84.4–99.5%) [58], while ofloxacin was more difficult to remove, and these residues were detected in wastewater at a higher rate than other types of antibiotics [49]. The improper disposal of antibiotics and medical waste in hospitals can contribute to the introduction of antibiotic residues in soil and underground water.

Most of the antibiotics administered to people in hospitals are used in homes and end up in domestic wastewater. Thus, municipal wastewater treatment plants (WWTPs) are one of the major sources of antibiotic-resistant bacteria (ARB) and ARGs released into the environment and have become a hotspot for HGT. Osinska et al. [59] showed a high potential for bacterially mediated HGT in wastewater environments. Single ARB are consistently associated with multiple ARGs. Once ARB successfully enter a WWTP, ARGs can be transmitted between the bacteria in the endogenous microbial community and the bacteria passing through the WWTP. Guo et al. [60] found that MGEs, including plasmids, transposons, integrons (intI1) and insertion sequences (e.g., ISSsp4, ISMsa21 and ISMba16) were abundant in sludge samples. Additionally, a network analysis indicated that some environmental bacteria might be potential hosts for multiple ARGs. Isolates resistant to β-lactams most frequently carried the *blaTEM* and *blaOXA* genes. The genomes that encode resistance to tetracyclines were most commonly *tetA*, *tetB* and *tetK*, while the *qnrS* gene was found in isolates resistant to fluoroquinolones [61]. Munir et al. [62] showed that the concentration of ARB decreased by several orders of magnitude compared to that in the original influent water, but the concentration of ARGs remained quite similar in pre- and post-disinfection effluents. There was no significant reduction in the abundance of MGEs in the effluent water either [63]. Compared with those in the original influent, most of the ARGs were effectively removed after wastewater treatment [64,65]. The specific environmental conditions in WWTPs offer a selective advantage for HGT of ARGs and ARB in bacterial communities.

The plasmid-mediated transfer of ARGs poses a grave danger to global public health. The use of amoxicillin on farms has made the poultry farm environment an essential reservoir of *blaNDM*-carrying bacteria [42,43]. Additionally, *blaNDM* contamination was also detected in the farm environment (soil, sewage, feed, dust) in commercial goose farms [66]. Moreover, IncX3- and pM2-1-type plasmids contribute to the prevalence and spread of ARGs in different bacteria. Mohsin et al. [44] detected IncFII- and IncQ-type plasmids carrying the *tet (X4)* gene in four different sources of *E. coli* (poultry, chicken, wild birds and slaughterhouse wastewater). In another study, all mcr-1-positive *E. coli* strains isolated from poultry were multidrug resistant, with up to 88.24% of the isolates containing *blaTEM* genes and tetracycline (*tetA* and *tetB*) and sulfonamide (*sulI*, *sulII* and *sulIII*) resistance genes [45]. The antibiotics commonly used in aquaculture are aminoglycosides, β-lactams, sulfonamides and tetracyclines [67]. Residual antibiotics leached from fish feed are often present in effluents. The levels of ARGs in fish farm effluents were found to be significantly higher than those in the surrounding water environment, and most of the ARGs were present on plasmids [68].

#### 4.2.2. Human Activities Affect the Transfer of ARGs in the Environment

The major dominant groups in agricultural sediments are *Actinobacteria*, *Chlamydomonas* and *Firmicutes* [69]. Wendi et al. [70] detected no antibiotic-associated resistance genes in aquaculture farm sediments used for farming, suggesting that natural resistance bodies may be present in farm sediments. However, the application of organic fertilizers to agricultural soils greatly contributes to resistance gene contamination. ARGs carried by bacteria in organic fertilizers and in antibiotics themselves have caused a significant increase in the abundance of resistance genes in fertilized soils [71,72]. Pu et al. [46] isolated two transferable amino-glycoside resistance plasmids from pig or chicken manure, namely, pRKZ3 and pKANJ7. As is known, pRKZ3 is a nonconjugated IncQ plasmid with *arr-3* and *aacA* resistance-conferring genes that encode plasmid replication and stabilization (*repA*, *repB* and *repC*) and mobilization (*mob*) functions. Furthermore, pKANJ7 is a conjugated IncQ plasmid encoding the T4SS-type IncX plasmid. Wang et al. [47] analysed the contamination of soil with ARGs in agricultural soils with long-term application of organic fertilizers. There is a high abundance of macrolide- and quinolone-resistant bacteria and drug resistance genes in fertilized soils in contrast to unfertilized soils. In addition, the abundances of intI and intII were significantly correlated with the abundances of *qnrS* and *ermB*, respectively. In general, intI is located on the Tn21 transposon, and intII is located on the Tn7 transposon, which has certain ramifications. Thus, this gene can be transmitted among bacteria via transposons. The *intl1* and *intl2* genes are frequently found in manure-treated agricultural soils and greenhouse soils. The broad availability of integrase genes can facilitate gene transfer, thereby increasing the persistence and accumulation of ARGs [73,74]. Zhao et al. [48] also found that the total relative abundance of the *intI* gene in manure-amended soil positively correlated with those of *tetW*, *tetO*, *sulI* and *sulII*. However, it has also been shown that the production of drug-resistant bacteria is negatively correlated with the dose of antibiotic exposure. This may be due to high antibiotic concentrations affecting the community structure and function of soil microorganisms. Some developed countries have applied sludge to agricultural production to reduce production costs [75]. The direct application of sludge also leads to the introduction of ARGs in agricultural systems. Markowicz [76] isolated 16 resistance genes and four integrator classes in sewage sludge containing plasmids with extreme resistance to β-lactams as well as tetracyclines. Iwu et al. [77] isolated multidrug-resistant *E. coli* containing plasmids harbouring AmpC and ESBLS in irrigation water and agricultural soil samples, as well as a plasmid-harbouring multigene sequence.

Talukder et al. [78] isolated multidrug-resistant *P. aeruginosa* from soils from industrial areas, and 60% of MARs carried 1000–2000 bp double plasmids, which suggests the occurrence of plasmid-mediated transfer of ARGs in industrial soils. This is most likely due to the targeted selection of resistant bacteria by certain concentrations of antibiotic residues. The horizontal transfer of ARGs in sediments is rarely reported compared to that in agricultural soils, but sediments are considered to be the main vector for the multiplication and translocation of antibiotics and ARGs [79]. Chen et al. [80] found that in the Pearl River basin, the *intI* and *sul* genes were dynamically transported between water and sediment, and *intI* was closely associated with some specific genes in the sediment [81]. Yang et al. [79] detected a higher variety and relative abundance of genes in the sediments of East Dongting Lake than in Hong Lake. Another study found that the most common ARGs in the coastal sediments of the East China Sea in China were sulfonamide resistance genes [82].

### 4.3. Transfer of ARGs under Other Selection Pressures

The co-selection of ARGs by heavy metals and antibiotics also increases ARG contamination in soil [83,84]. Xu et al. [85] reported correlations between heavy metals and some ARG subtypes and observed positive correlations between Zn and the *intI* gene, with Cu and Zn having stronger positive correlations with ARGs than antibiotics. This implies that metals may play an important role in increasing the integration frequency of ARGs in various bacteria in agricultural soils. Both copper oxide nanoparticles and copper ions (Cu^2+^) can facilitate the conjugative transfer of multiple resistance genes [86]. Heavy metal exposure accelerates the plasmid-mediated conjugative transfer of ARGs. Although nanomaterials can remove heavy metals by adsorption, Cd^2+^ and high concentrations of Fe_2_O_3_ nanoparticles significantly increase the frequency of the conjugative transfer of RP4 plasmids [87]. High concentrations of metals in soil affect the composition and function of soil bacterial communities. Klumper et al. [88] demonstrated for the first time that metal stress can modulate the tolerance of different soil bacteria to IncP plasmids. Soil minerals also affect the rate of the conjugative transfer of plasmids carrying ARGs, and the effect of different types of soil minerals on the rate of conjugative transfer varies [89]. Herbicides can cause changes in the susceptibility of certain strains to antibiotics and can also accelerate the HGT of ARGs in soil bacteria [90]. It has been shown that herbicide-use has a weak effect on the abundance and composition of soil microbial communities but can increase the abundance of corresponding ARGs and MGEs as well as the coupling frequency of plasmids [91].

## 5. Phage-Mediated Transfer of Antibiotic Resistance Genes

Phages can transfer genes by specific or universal transduction. Specific transduction involves the transfer of only a few specific genes, whereas universal transduction can move any segment of the bacterial genome. Another mechanism that is similar to transduction but different in nature is lysogenic conversion. When a mild phage infects a host bacterium, the phage DNA integrates with the host chromosome, causing the host to become lysogenic and leading it to acquire certain characteristic traits. Certain phenotypes of the host can also be altered by lysogenic transformation, leading to the acquisition or loss of a trait. Among several mechanisms of DNA transfer, lysogenic transformation caused by phage is more dominant and efficient [92]. Once phage-transferred ARGs reach the recipient bacteria by either mechanism, the survival of ARGs depends on the ability of the sequence to integrate into the bacterial genome. If ARGs are specifically transduced by phage transfer, an intact phage genome including the integrase gene will increase the chances of successful integration. If the gene is transduced by universal transduction, then the successful transfer of ARGs requires the recombination of the exogenous gene into the host chromosome. Thus, the genes encoding recombinase and integrase will determine the efficiency of the acquisition of ARGs by the recipient bacterium [93]. The presence of phages in aqueous environments and their potential for the HGT of ARGs have been widely demonstrated [94], but has been less studied in soil environments. Blance [95] et al. isolated phage particles carrying five ARGs (*bla_TEM_*, *bla_CTX-M-1_*, *bla_CTX-M-9_*, *sul1* and *tetW*) from seawater. Another study found that fluoroquinolone exposure of multidrug-resistant *Salmonella* induced its phage-mediated gene transfer [96]. However, several studies have found phages carrying ARGs in the faces of poultry, cattle, pigs and even humans [97,98]. In manure-amened agricultural soils, this undoubtedly gives rise to a significant risk of phage-mediated transfer of ARGs.

## 6. One Health Approach of Antibiotics Resistance

The United Nations has set the goal of “Good Health and Well-being” to ensure healthy lives and to promote well-being for all at all ages [99]. However, the use of antibiotics in humans, livestock farming, and agricultural lands has led to significant environmental stress, which in turn has contributed to the prevalence of antibiotic resistance. As a large agricultural country, China undoubtedly has a great risk of antibiotic contamination in the soil environment and in the spread of ARGs. The application of animal manure with high levels of residual antibiotics, ARB and ARGs increases the risk of introducing ARGs into agricultural soils [100,101]. In manure-amended soils, increased antibiotic concentrations and the associated abundance of resistance genes are accompanied by enhanced correlations between class I integrons and ARGs [102].

In recent years, phytochemicals such as alkaloids and phenolic compounds have been shown to be alternatives to traditional antibiotics for the treatment of infections caused by corresponding antibiotic-resistant bacterial pathogens. The sephytochemicals act on membrane proteins, biofilms, efflux pumps and other structures closely related to gene transfer at the level of ARGs, thus inhibiting the growth of resistant bacterial pathogens [103]. Functional antimicrobial peptides (AMPs) are an important class of effector molecules for the innate host immune defense against pathogen invasion. AMPs (cecropin A and melittin) extracted from insects do not induce stress pathways in bacteria. *Hermetia illucens* AMPs have been demonstrated to have the potential to replace antibiotics in animal husbandry [104].

## 7. Outlook

In bacteria, the HGT of ARGs is mainly carried out through MGEs such as phages and plasmids. Phage-mediated HGT occurs mainly within species because phage transmission is limited by the genetic similarity of hosts, but plasmids can cross interspecies barriers, and the HGT mediated by plasmids has a larger range and higher frequency [105]. Invasive bacteria can carry plasmids into plant and animal cells, plasmids can be integrated into the genome for stable expression in daughter cells, and some chromosomal plasmids can even be vertically transferred with the bacteria carrying them. The plasmid-binding-related transfer mechanism has now been demonstrated in model plasmids, but studies on the presence and nature of potential signals for activating splice pairing have yet to be addressed. Not only can plasmids mediate the HGT of antibiotic resistance, but other virulence genes and adaptors are also applicable. Although studies have been conducted to investigate how HGT promotes the transmission, persistence, and maintenance of virulence of pathogenic bacteria through whole-genome sequencing data, the scope of such studies is relatively narrow [106,107]. For mobile ARGs, most studies have focused only on specific classes of ARGs, such as sulfonamide resistance genes and tetracycline resistance genes, and there is a lack of systematic generalized analyses on the general migration and transformation mechanism of ARGs. The contribution of soil plasmids to the spread of resistance genes needs to be further investigated as drug-resistant bacteria spread globally and the understanding of phages improves.

Bacteria are involved in HGT as vectors for the spread of ARGs in different environments (sewage sludge, manure, agricultural soil, etc.), posing a great threat to the natural environment and human social life. Plasmid-bacteria interactions are extremely complex, and even multidrug-resistant bacteria are commonly observed, so the effective prevention of the transmission of resistance genes through the plasmid-bacteria pathway needs to be further explored. There are more studies on the transmission mechanisms of ARGs in aquatic environments, including the linkage of ARGs between primitive polar glaciers and urban rivers or coastal seas. The transport and transmission of ARGs between soils and plant bodies has also been reported, but the transport pathways of ARGs between aqueous and soil environments or even atmospheric environments have been less well studied. Mucin is the last drug used in the treatment of Gram-negative infections, and further studies on the plasmid-mediated genes of resistance to mucin should be performed. When grown on antibiotic-contamination soils with a high abundance of resistance genes, the products eventually move through the food chain to the next level of consumers, thus forming a chain of resistance-gene transmission. Tracking studies for a specific class of ARGs to characterize the entire cycle is a worthy direction for future research.

## Figures and Tables

**Table 1:** Distribution of antibiotic resistance genes in different environments.

ARGs	Antibiotic Types	Origin	References
nonmobile dihydropteroate synthase (DHPS) genes	sulfonamides	Beech and pine forest soils	[33]
*qnrB*, *aacC*, *blaOXY*, *sulI*, *sulII*, *sulIII*, *tetD*, *tetA*, *tetM01*, *tetW*, *tetR*	quinolone, aminoglycoside, beta-lactam, sulfonamide, tetracycline	Primeval forest soil	[34]
*tetA*, *tetL*, *addD*, *merA*, *bla_SHV_*	aminoglycoside, sulfonamides, tetracycline	Manure-amended agricultural soil	[35]
*aadA*, *acrA*, *ampC*, *bla_TEM_*, *bla_CTX_*, *ermC*, *vanTC*, *vanRA*, *tetT*, *tetL*	aminoglycoside, beta-lactam, sulfonamides, tetracycline, vancomycin	Greenhouse vegetable production bases	[36]
*tetA*, *tetQ*, *tetX*, *tetM*, *bla_TEM_*, *sul1*, *sul2*, *strB*, *qnrS*, *ermB*, *ermC*, *oqxB*, *cfr*	quinolone, beta-lactam, sulfonamide, tetracycline, chloromycetin, streptomycin	Layer farm soil	[37,38]
*rpoB2*, *rpoB*, *rphA*, *mdtB*, *mdtC*, *vanRO*	rifamycin, aminocoumarin, glycopeptide	Arctic permafrost zone	[39]
*carA*, *macB*, *bcrA*, *taeA*, *srmB*, *tetA*, *oleC*, *sav1866*, *tlrC*	macrolides, glycopeptides, tetracyclines	Deep-sea sediments	[40,41]
*blaNDM*, *blaTEM*, *tet (X4)*, *tetA*, *tetB*, *sulI*, *sulII*, *sulIII*	beta-lactam, tetracycline, sulfonamides	Farm, Aquaculture wastewater	[42,43,44,45]
*arr-3, aacA, qnrS, ermB, tetW, tetO, sulI, sulII*	aminoglycoside, macrolides, quinolone, tetracycline, sulfonamide	Domestic wastewater, Medical wastewater	[46,47,48,49,50]

## Data Availability

Not applicable.
